# Biochemical and structural characterization of C-terminal constructs of bovine soluble guanylate cyclase

**DOI:** 10.1186/1471-2210-11-S1-P67

**Published:** 2011-08-01

**Authors:** Franziska Seeger, Elsa D Garcin

**Affiliations:** 1 Department of Chemistry Biochemistry, University of Maryland Baltimore County, Baltimore MD 21250, USA

## Background

Soluble guanylate cyclase (sGC) is the key enzyme in the NO-sGC-cGMP signaling cascade crucial in regulating the cardiovascular system. Low output of this system causes hypertension and acute heart failure, which are the leading causes of death globally.

Mammalian sGC is a heterodimer. Each of the two homologous subunits (α and β) contains three domains: an N-terminal regulatory domain (HNOX: Heme Nitric oxide OXygen), a central dimerization HNOX associated (HNOXA) and coiled-coil (CC) domain, and a C-terminal catalytic domain (GC).

The enzyme is basally active, but NO binding to the heme group in the β subunit’s regulatory domain enhances sGC catalytic output several hundred fold. The exact mechanism by which the regulatory domain relays the NO activation signal to the catalytic domain remains elusive. Furthermore, it has been proposed that the HNOX regulatory domain inhibits the activity of the catalytic GC domain [1]. Winger *et al.* showed that the GC heterodimer by itself exhibits catalytic activity in the presence of Mg^2+^ and Mn^2+^[1]. On the contrary, Wedel *et al.* propose that additional amino acids are required for dimerization, folding, and catalytic activity [2].

We aim to test the hypothesis that additional domains are necessary for full activity of the catalytic domain by combining mutagenesis, activity assays, fluorescence spectroscopy, Small-Angle X-ray Scattering (SAXS), and protein crystallography.

## Results

**Figure 1:**
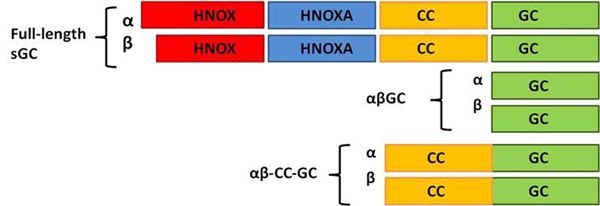
Full-length sGC and C-terminal truncated constructs αβGC and αβCC-GC.

We have chosen a divide-and-conquer approach to study sGC catalysis. Here, we report the recombinant expression and purification of bovine C-terminal constructs “αβGC” and “αβCC-GC” in *E. coli* (Figure 1). Preliminary activity measurements for these constructs in the presence of Mg^2+^ show that αβCC-GC exhibits higher level of catalytic activity than αβGC (4-fold). This suggests that additional domains are necessary for full catalytic activity. To our knowledge this is the first report that attributes catalytic activity to the αβCC-GC heterodimer in the presence of Mg^2+^.

## Conclusion

The C-terminal sGC constructs αβGC and αβCC-GC, both exhibit catalytic activity in the presence of Mg^2+^. Higher levels of activity of αβCC-GC as compared to αβGC hint at structural differences between the two constructs that will be characterized using protein X-ray crystallography and small-angle X-ray scattering.
